# Locoregionally Recurrent Colon Cancer: How Far Have We Come? A Population-Based, Retrospective Cohort Study

**DOI:** 10.1245/s10434-022-12689-5

**Published:** 2022-10-19

**Authors:** Hidde Swartjes, Daan W. P. van Lankveld, Felice N. van Erning, Henk M. W. Verheul, Johannes H. W. de Wilt, Tijmen Koëter, Pauline A. J. Vissers

**Affiliations:** 1 Department of Surgery, Radboud Institute for Health Sciences, Radboud University Medical Center, Nijmegen, The Netherlands; 2 Department of Research and Development, Netherlands Comprehensive Cancer Organization, Utrecht, The Netherlands; 3 Department of Surgery, Catharina Hospital, Eindhoven, The Netherlands; 4 Department of Medical Oncology, Radboud Institute for Health Sciences, Radboud University Medical Center, Nijmegen, The Netherlands; 5 Department of Surgery, Elisabeth-TweeSteden Hospital, Tilburg, The Netherlands

## Abstract

**Background:**

The reported outcomes of locoregionally recurrent colon cancer (LRCC) are poor, but the literature about LRCC is scarce and aged. Recent population-based studies to provide current insight into LRCC are warranted. This study aimed to provide an overview of the incidence, risk factors, treatment, and overall survival (OS) of patients with LRCC after curative resection of stage I–III primary colon cancer.

**Methods:**

Data on disease recurrence were collected for all patients with a diagnosis of non-metastasized primary colon cancer in the Netherlands during the first 6 months of 2015. Patients who underwent surgical resection (N = 3544) were included in this study. The 3-year cumulative incidence, risk factors, treatment, and OS for patients with LRCC were determined.

**Results:**

The 3-year cumulative incidence of LRCC was 3.8%. Synchronous distant metastases (LRCC-M1) were diagnosed in 62.7% of the patients. The risk factors for LRCC were age of 70 years or older, pT4, pN1-2, and R1-2. Adjuvant chemotherapy was associated with a decreased risk of LRCC for high-risk stage II and stage III patients [hazard ratio (HR), 0.47; 95% confidence interval (CI) 0.31–0.93]. The median OS for the patients with LRCC was 13.1 months (95% CI 9.1–18.3 months). Curative-intent treatment was given to 22.4% of the LRCC patients, and the subsequent 3 years OS was 71% (95% CI 58–87%). The patients treated with palliative treatment and best supportive care showed 3-year OS rates of 15% (95% CI 7.0–31%) and 3.7% (95% CI 1.0–14%), respectively.

**Conclusions:**

The cumulative incidence of LRCC was low, and adjuvant chemotherapy was associated with a decreased risk for LRCC among targeted patients. Curative-intent treatment was given to nearly 1 in 4 LRCC patients, and the OS for this group was high.

**Supplementary Information:**

The online version contains supplementary material available at 10.1245/s10434-022-12689-5.

Colorectal cancer (CRC) is the second leading cause of cancer death and the third most commonly diagnosed cancer.^1^ In approximately two thirds of CRC cases, the tumor is located in the colon.^2^

Curative treatment of colon cancer is based primarily on tumor resection, followed by adjuvant chemotherapy for high-risk stage II and stage III patients.^3,4^ Due to advancements in treatment strategies, the survival of patients with primary colon cancer has improved significantly during the last decades.^3,5^

Still, 13–16% of patients with stage I–III primary colon cancer experience a recurrence of colon cancer within the first 3 years after treatment.^6^ Most recurrences are distant (e.g., located in the liver or lungs), but locoregionally recurrent colon cancer (LRCC) is diagnosed in approximately 4–12% of patients who have undergone curative resection of stage I–III primary colon cancer.^7–13^

The risk for the development of LRCC is higher for more advanced primary tumors, as well as for patients with an incomplete resection margin.^8,10,12,13^ The outcomes of LRCC are poor, with a reported median overall survival (OS) of approximately 9 months after diagnosis.^8^ However, the 5-year OS increases to approximately 50% when a resection of LRCC with clear margins is reached.^14^

Previously published studies of LRCC are based predominantly on data from more than 10 years ago, all acquired from selective geographic regions or single centers.^14,15^ Whereas the literature on the occurrence and treatment of locally recurrent rectal cancer is extensive, the data on LRCC are scarce. Moreover, previously conducted studies of treatment strategies for LRCC and their corresponding survival outcomes are constrained by a relatively small number of cases. Obtaining insight into current treatment strategies and corresponding survival outcomes is necessary because it could enhance standardization of LRCC management and thereby positively influence LRCC outcomes.

Using recent population-based data from the Netherlands Cancer Registry (NCR), this study aimed to provide an overview of the incidence, risk factors, treatment, and OS of LRCC among patients with diagnosed with stage I-III primary colon cancer in the Netherlands during the first 6 months of 2015 who were subsequently treated with surgical resection.

## Methods

### Study Design

Patient, tumor, treatment, and survival data were obtained from the Netherlands Cancer Registry (NCR). All cases of newly diagnosed primary colorectal cancer (CRC) in the Netherlands have been included in this registry since 1989. Patient, tumor, and treatment data were extracted from electronic patient files in all hospitals in the Netherlands by trained data managers. Pathologic data were retrieved from the automated national pathologic archive (PALGA). Data on OS were available through annual linkage of the NCR with the Dutch personal records database.

Follow-up assessment of vital status for this study was complete up to 1 February 2021. The NCR does not include data on ethnicity and race. The conduct of this study was approved by the ethics committee of the NCR.

Data on recurrences of colon cancer are not regularly registered in the NCR. By exception, clinical follow-up data were collected for the cohort of patients diagnosed with non-metastasized primary colon cancer during the first 6 months of 2015 (i.e., from 1 January to 30 June). This additional data collection was performed in 2019. Tumor and treatment data on the first CRC recurrence were subsequently extracted from electronic patient files and registered in the NCR. Therefore, the cohort with pathologic stage I-III primary colon cancer cases diagnosed in the Netherlands between 1 January and 30 June of 2015 represented the base population for the present study.

Patients with appendiceal tumors, neuro-endocrine tumors, pT0 tumors, and endoscopically resected tumors were excluded from this study. If patients presented with more than one colonic tumor simultaneously, the tumor with the lowest stage was excluded.

Tumor location and morphology were coded according to the International Classification of Disease-Oncology (ICD-O-3). Tumor location was categorized as right colon (C18.0, C18.2–C18.4), left colon (C18.5–C18.7), overlapping (C18.8), or unknown (C18.9). Morphology was categorized as non-mucinous adenocarcinoma (8140–8389), mucinous adenocarcinoma (8470 and 8480), signet cell carcinoma (8490) and other. Clinical and pathologic tumor staging was registered according to the Union for International Cancer Control (UICC) tumor-node-metastasis (TNM) classification (7th edition). An incomplete resection (R1) was defined as a resection margin smaller than 1 mm.

Locoregionally recurrent colon cancer was defined as recurrence near the site of the primary tumor, recurrence in lymph nodes that would classify as regional lymph nodes according to the TNM classification, or both. No data on the exact location of the locoregional recurrence were collected. For specific analyses, LRCC was subdivided into LRCC diagnosed without synchronous distant metastases (LRCC-M0) and LRCC diagnosed with synchronous distant metastases (LRCC-M1) according to the TNM classification.

Systemic therapy was defined as chemotherapy, targeted therapy, or both. Curative-intent treatment was defined as resection of the LRCC in LRCC-M0 patients and as LRCC resection with metastasectomy or radiofrequency ablation (RFA) in LRCC-M1 patients. Palliative treatment was defined as systemic therapy, radiotherapy, and/or metastasectomy/RFA in the absence of LRCC resection. Best supportive care was defined as the absence of both surgical and non-surgical treatment.

This study was listed in the ClinicalTrial.gov registry (NCT05475288).

### Statistical Analyses

If a patient dies before the development of LRCC, death is a competing risk to a diagnosis of LRCC. Likewise, as only the first recurrence was registered, the diagnosis of isolated distant recurrences was another competing risk to an LRCC diagnosis in these data. In the situation of competing risks, competing risk analyses are superior to the more general Kaplan–Meier and Cox regression analyses.^16^ Therefore, the cumulative incidence of LRCC was estimated using competing risk cumulative incidence analyses, and between-group differences were compared using Gray’s test.

Covariates were assessed for their association with LRCC using uni- and multivariable competing risk regression analyses with complete cases according to the cause-specific hazard approach.^17^ Covariates were included in the multivariable model if *p* was lower than 0.10 in univariable analyses. To handle missing data and prevent selection bias, covariates with more than 5% missing values were not included in the main multivariable model. Multiple imputation was not possible because the missing data were most likely not missing at random, but were more likely present due to logistic reasons in several hospitals. An exploratory multivariable model in which covariates with more than 5% missing data and a *p* value lower than 0.10 in the univariable analysis were included, was presented alongside the main multivariable model to be enable weighing of the outcomes from the main multivariable model. However, these results should be interpreted with caution due to the increased risk for selection bias. The association between administration of adjuvant chemotherapy and LRCC risk, adjusted for age, was determined using multivariable analyses of pT4N0 (i.e., high-risk stage II) and pT1-4N1-2 (i.e., stage III) patients, because these patients qualified for adjuvant treatment according the previous and most recent treatment guidelines.^18,19^

Overall survival was estimated using the Kaplan–Meier method and compared using the log-rank test. A *p* value lower than 0.05 was regarded as indicating statistical significance. All statistical analyses were performed using R version 4.1.3 in combination with the “survival” and “cmprsk” package.

## Results

### Primary Tumor Characteristics

Of the 4123 cases of primary colon cancer in the base study population, 558 did not meet the inclusion criteria that specified non-surgical resection (*n* = 528), no pathologic stage I-III colon cancer (*n* = 18), and tumor localization in the appendix (*n* = 12). Additionally, 11 patients presented with multiple colonic tumors. For these 11 patients, the tumor with the lowest stage was excluded. Consequentially, 3554 patients were included in the study (Table 1).

**Table 1:** Characteristics of patients and primary colon cancer

Characteristics	*n* (%)
Age: years (IQR)	69 (63–76)
*Sex*	
Male	1931 (54.3)
Female	1623 (45.7)
*Location*	
Right colon	1663 (46.8)
Left colon	1854 (52.2)
Overlapping	24 (0.7)
Unknown/missing	13 (0.4)
*Pathologic cancer stage*	
I	936 (26.3)
II	1356 (38.2)
III	1262 (35.5)
*Pathologic tumor stage*	
pT0	1 (0.0)
pT1	449 (12.6)
pT2	657 (18.5)
pT3	1959 (55.1)
pT4	486 (13.7)
Unknown/missing	2 (0.1)
*Pathologic nodal stage*	
pN0	2282 (64.2)
pN1	833 (23.4)
pN2	429 (12.1)
Unknown/missing	10 (0.3)
*Resection margin*	
R0	3470 (97.6)
R1-2	53 (1.5)
Unknown/missing	31 (0.9)
Pathologically assessed lymph nodes (IQR)	17 (13–24)
*Morphology*	
Non-mucinous adenocarcinoma	3137 (88.3)
Mucinous adenocarcinoma	357 (10.0)
Signet cell carcinoma	34 (1.0)
Other	26 (0.7)
*Lymphovascular invasion*	
No	2284 (64.3)
Yes	844 (23.7)
Unknown/missing	426 (12.0)
*Differentiation grade*	
Good	116 (3.3)
Moderate	2772 (78.0)
Poor	337 (9.5)
None	4 (0.1)
Unknown/missing	325 (9.1)
*Adjuvant chemotherapy*	
No	2531 (71.2)
Yes	1023 (28.8)

*IQR* interquartile range

The median age at onset of primary colon cancer was 69 years (interquartile range [IQR], 63–76 years). More male than female primary colon cancer patients were included (54.3% vs 45.7%, respectively). The majority of the primary colon cancer patients had microscopically complete resection margins (97.6%). Adjuvant chemotherapy was administered to 0.6%, 11.7% and 87.7% of pathologically staged I, II and III primary colon cancer patients.

### Incidence of LRCC

The median clinical follow-up time after primary colon cancer resection was 43.1 months (IQR 35.4–47.2 months). Of the 134 patients diagnosed with LRCC, 50 (37.3%) presented without synchronous distant metastases (LRCC-M0), and 84 (62.7%) presented with synchronous distant metastases (LRCC-M1). The LRCC was classified as local for 79 patients (59.0%), as regional for 35 patients (26.1%), and as a combination of local and regional for 20 patients (14.9%). The median age at LRCC diagnosis was 72 years (IQR 66–80 years).

**Fig. 1:**
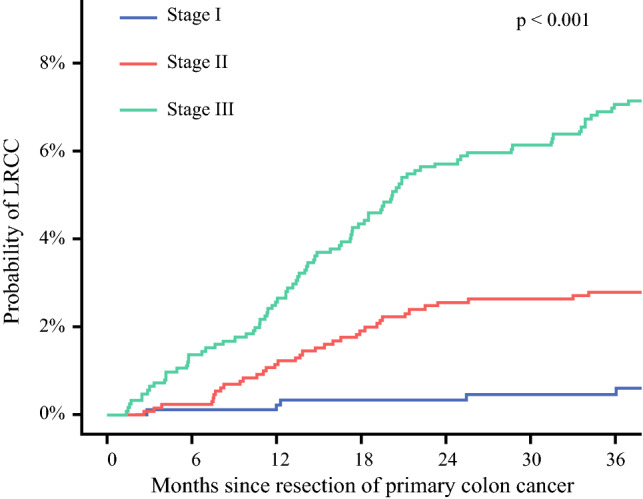
Cumulative incidence plot of locoregionally recurrent colon cancer (LRCC) stratified for pathologic stage of primary colon cancer

The cumulative incidence of LRCC was 1.4% at 1 year and 3.8% at 3 years. The 3-year cumulative incidence of LRCC was 0.5% for pathological stage I, 2.8% for pathological stage II, and 7.0% for pathological stage III primary colon cancer (*p* < 0.001; Fig. 1).

**Fig. 2:**
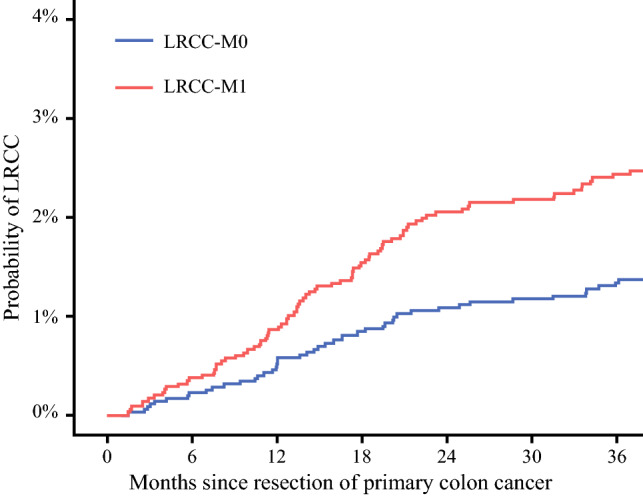
Combined cumulative incidence plot of LRCC-M0 and LRCC-M1. *LRCC* locoregionally recurrent colon cancer

The 3-year cumulative incidence of LRCC-M0 was 1.3%, while 3-year cumulative incidence of LRCC-M1 was 2.4% (Fig. 2). The majority of the LRCC-M1 patients presented with synchronous metastases in one organ/site (*n* = 46, 58.4%). Synchronous metastases in two, three, or four organs/sites were present in 19 (22.6%), 12 (14.3%), and 7 (8.3%) LRCC-M1 patients, respectively.

### Risk Factors for LRCC

In the multivariable analyses, an increased risk for LRCC was associated with age of 70 years or older at onset of primary colon cancer (HR, 1.7; 95% CI 1.2–2.5; *p* = 0.002), pT4 tumors (HR 4.1; 95% CI 2.8–6.0; *p* < 0.001), pN1–2 tumors (HR 2.9; 95% CI 2.0–4.3; *p* < 0.001), and an incomplete resection margin (HR 5.5; 95% CI 3.2–9.5; *p* < 0.001) (Table 2, *n* = 3506 included cases). Addition of lymphovascular invasion and differentiation grade to this multivariable analysis excluded 699 additional cases from the model (*n* = 2807 included cases; Table S1) but led to the identification of both lymphovascular invasion (HR 2.0; 95% CI 1.3–2.9; *p* = 0.002) and poor to no tumor differentiation (HR 1.9; 95% CI 1.3–2.9; *p* = 0.004) as independently associated with an increased risk for LRCC.

**Table 2:** Uni- and multivariable competing risk regression output for the risk of locoregionally recurrent colon cancer (LRCC) according to the cause-specific hazard method

	3 Years LRCC estimate (%)	Univariable HR (95% CI) (*n* = 3554)	*p* value^a^	Multivariable HR (95% CI) (*n* = 3506)	*p* value^a^
*Age (years)*					
< 70	2.9	Reference		Reference	
≥ 70	4.7	1.7 (1.2–2.4)	0.002	1.7 (1.2–2.5)	0.002
*Sex*					
Male	3.7	Reference			
Female	3.8	1.0 (0.72–1.4)	0.960		
*Location*					
Right colon	4.2	Reference			
Left colon	3.5	0.81 (0.58–1.1)	0.228		
*Pathologic T stage*					
pT1-3	2.2	Reference		Reference	
pT4	13.3	7.2 (5.1–10.1)	< 0.001	4.1 (2.8–6.0)	< 0.001
*Pathologic N stage*					
pN0	1.8	Reference		Reference	
pN1-2	7.0	4.2 (2.9–6.0)	< 0.001	2.9 (2.0–4.3)	< 0.001
*Resection margin*					
R0	3.2	Reference		Reference	
R1-2	32.1	14.3 (8.6–23.8)	< 0.001	5.5 (3.2–9.5)	< 0.001
*No. of assessed lymph nodes*					
< 10	3.1	Reference			
≥ 10	3.8	1.3 (0.59–2.7)	0.537		
*Morphology*					
Non-mucinous adenocarcinoma	3.6	Reference			
Mucinous adenocarcinoma, signet cell carcinoma, and other	4.8	1.3 (0.83–2.1)	0.232		

*HR* hazard ratio, *CI* confidence interval

Covariate selection for the multivariable model was based on *p* < 0.10 in univariable analyses

^a^*p* Values lower than 0.05 were regarded as indicating statistical significance

Adjuvant chemotherapy was administered to 955 (65.7%) of 1453 patients with high-risk stage II or III primary colon cancer. In these patients, adjuvant chemotherapy after curative resection of primary colon cancer was associated with a decreased risk for LRCC (HR, 0.47; 95% CI 0.31–0.93; *p* < 0.001; *n* = 1451 included cases) after adjustment for age. This association could not be proven for high-risk stage II patients only (HR, 0.39; 95% CI 0.12–1.3; *p* = 0.114; *n* = 191 included cases), but was present in stage III patients (HR, 0.52; 95% CI 0.33–0.83; *p* = 0.006; *n* = 1260 included cases).

### Treatment of LRCC

**Fig. 3:**
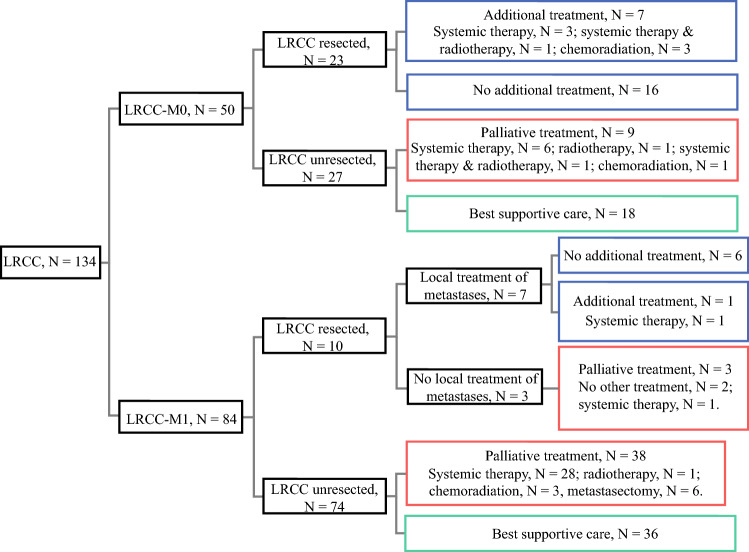
Treatment of LRCC-M0 and LRCC-M1 patients. *Blue box:* curative-intent treatment. *Red box:* palliative treatment. *Green box:* best supportive care. *LRCC* locoregionally recurrent colon cancer

Curative-intent treatment was given to 22.4% of the LRCC patients. Palliative treatment was administered to 37.3% and best supportive care to 40.3% of the LRCC patients (Fig. 3).

The median age at diagnosis of LRCC in both the curative-intent and palliative treatment groups was 69 years, while the median age at diagnosis of LRCC was 80 years in the best supportive care group (*p* < 0.001). Proportionally, the LRCC-M0 patients received curative-intent treatment more often than the LRCC-M1 patients (46.0% vs 8.3%; *p* < 0.001).

### Survival with LRCC

The median survival after diagnosis of LRCC, irrespective of synchronous distant metastases and treatment, was 13.1 months (95% CI 9.1–18.3 months). Overall survival was 53% (95% CI 45–62%) at 1 year and 27% (95% CI 20–35%) at 3 years after diagnosis of LRCC.

**Fig. 4:**
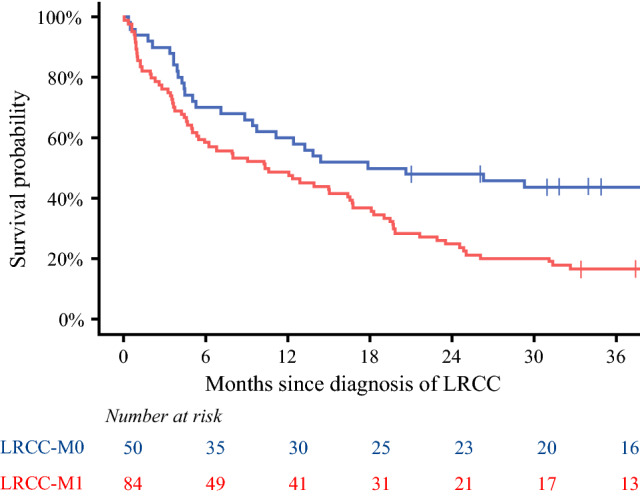
Overall survival plot stratified for LRCC-M0 and LRCC-M1. *LRCC* locoregionally recurrent colon cancer

The median survival for the LRCC-M0 patients was 19.3 months (95% CI 9.7 months-upper value not reached). OS for the LRCC-M0 patients was 60% (95% CI 48–75%) at 1 year and 44% (95% CI 32–60%) at 3 years after diagnosis of LRCC. The LRCC-M1 patients had a median survival time of 10.4 months (95% CI 5.5–16.7 months), a 1-year OS of 49% (95% CI 39–61%), and a 3-year OS of 17% (95% CI 10–27%). The LRCC-M0 patients showed a significantly better OS than the LRCC-M1 patients (*p* = 0.002; Fig. 4).

The 49 patients with a diagnosis of LRCC during the first year after resection of primary colon cancer had a worse median OS (6.8 months; 95% CI 4.4–14.4 months) than the 85 patients with a diagnosis of LRCC subsequent to the first year after resection (median OS, 18.1 months; 95% CI 12.3–25.0 months; *p* = 0.005).

**Fig. 5:**
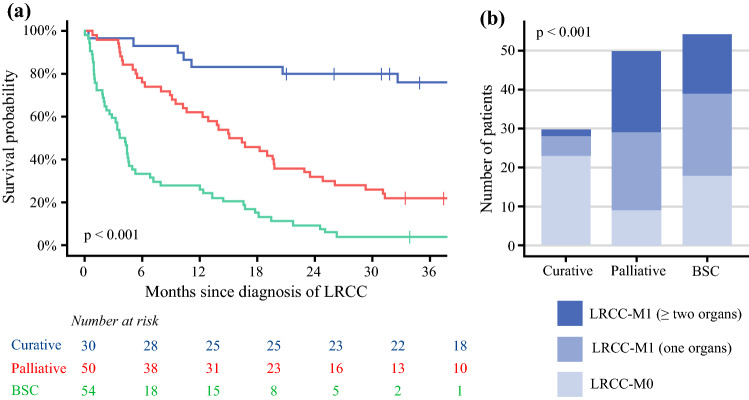
Overall survival plot stratified for treatment groups (**a**) complemented by a stacked bar chart displaying the distribution of LRCC-M0 patients, LRCC-M1 patients with synchronous metastases in one organ/site only, and LRCC-M1 patients with synchronous metastases in two or more organs/sites within and across the treatment groups (**b**). *LRCC* locoregionally recurrent colon cancer, *BSC* best supportive care

The 3-year OS after curative-intent treatment was 71% (95% CI 58–87%; Fig. 5A). This group consisted mainly of LRCC-M0 patients (76.7%; Fig. 5B). The palliatively treated LRCC patients showed a 3-year OS of 15% (95% CI 7.0–31%). The LRCC patients who had received best supportive care showed a 3-year OS of 3.7% (95% CI 1.0–14%).

## Discussion

The present large population-based study showed that LRCC in patients with surgically resected stage I–III primary colon cancer is relatively rare, with a 3-year cumulative incidence of 3.8%. In the multivariable analysis, age of 70 years or older at onset, locally advanced tumor and nodal stage, and positive resection margins were associated with a higher risk for LRCC. Two thirds of the LRCC patients presented with synchronous distant metastases. This group showed a significantly worse OS than the LRCC-M0 patients. The curatively treated LRCC patients (i.e., LRCC resection for the LRCC-M0 patients and LRCC resection with metastasectomy/RFA for the LRCC-M1 patients) showed a high 3-year OS of 71%.

Two population-based studies previously estimated the cumulative incidence of LRCC using the Kaplan–Meier method.^8,10^ In the present study, the competing risk cumulative incidence method was used, which is methodologically superior to the Kaplan–Meier method in the situation of competing events.^16^ Still, estimates resulting from both methods can be compared with each other. Sjövall et al.^8^ estimated the 5-year cumulative incidence of LRCC to be 11.5% in a Swedish population-based cohort treated for primary colon cancer between 1996 and 2000. Elferink et al.^10^ used population-based data of primary colon cancer patients diagnosed between 2000 and 2003 from the Netherlands to estimate the 5-year cumulative incidence at 6.4%.

The 3-year cumulative LRCC incidence of 3.8% estimated in the present study likely will result in a lower 5-year cumulative incidence than that reported by Sjövall et al.^8^ because the vast majority of recurrences occur within the first 3 years after treatment.^20^ This low nationwide incidence of LRCC might be explained by improved perioperative and surgical care due to centralization, multidisciplinary team meeting discussions of all CRC patients, and auditing of outcomes during the last decade in the Netherlands.^3^

An interesting finding was that right- and left-sided localizations had very comparable LRCC cumulative incidence rates and did not differ significantly in the univariable competing risk regression analysis. Liska et al.^13^ found similar results, whereas Elferink et al.^10^ identified left-sided primary colon cancer as a risk factor for LRCC. Right-sided location was identified as a risk factor by Park et al.^12^ and Sjövall et al.^8^ found locations of the splenic flexure and the sigmoid colon to be an independent risk factor for LRCC. The exact role of primary colon cancer location in the incidence of LRCC remains controversial.

Due to the high proportion of missing values of lymphovascular invasion and differentiation grade, these covariates could not be included in the main multivariable model. Nonetheless, the exploratory multivariable analysis including these covariates led to the exclusion of 21% of the main cohort, but suggests a significant influence of LRCC risk. Subsequent studies should focus on these possible associations because no definite conclusions can be drawn from these data due to a risk of selection bias. Another disputable risk factor for LRCC is the administration of adjuvant chemotherapy during treatment of the primary tumor. Liska et al.^13^ concluded that adjuvant chemotherapy did not show an advantageous effect on LRCC regardless of primary colon cancer staging after Kaplan–Meier analyses. In contrast, Elferink et al.^10^ showed that the absence of adjuvant chemotherapy was a risk factor for LRCC; this finding was underlined by the multivariable analyses for high-risk stage II and stage III tumors in the present study. This association could not be proven for high-risk stage II patients only, but the combination of a low HR of 0.39 and the small sample size of 191 cases suggests that this was due to a lack of statistical power of this analysis.

Previous studies reporting on outcomes of curative-intent treatment for LRCC used study populations containing between 3 and 31% of LRCC-M1 patients,^21–25^ in accordance with the 23.3% of LRCC-M1 patients (*n* = 7/30) in the present study. Despite this noticeable proportion of LRCC-M1 patients, the 3 years OS after curative-intent treatment was high (71%). This statistic can be considered excellent in comparison with the literature.

The largest and most recent systematic review with meta-analysis from 2016 on treatment of LRCC reported a pooled 3-year OS of 58% after R0 resection of LRCC.^14^ The study by Sjövall et al.^8^ published in 2007 using data from 1996 to 2000, was the only other population-based study that reported on OS for patients with LRCC. The 5-year OS after R0 resection was estimated to be 43%, and the corresponding Kaplan–Meier curve portrayed a 3-year OS between 50 and 55%.

A recent Canadian multicenter cohort study found a 5-year OS of 75% in a LRCC population comprising only a 4% proportion of LRCC-M1 patients.^21^ The results from the Canadian multicenter study and our population-based study illustrate that the OS after curative-intent treatment of LRCC in recent years is high. This high OS is most likely due to an adequate selection of patients for curative-intent treatment during multidisciplinary team meetings attended by, among others, medical oncologists, surgeons, anesthesiologists, and geriatricians. These multidisciplinary team meetings have become the gold standard for establishing the treatment course of challenging colorectal cancer cases in the Netherlands.^26^

The role of chemotherapy and radiation therapy in the curative-intent treatment of LRCC is unclear. This is reflected by the absence of a nationwide guideline recommendation on the treatment of LRCC during the inclusion period of the present study. Previous studies showed great heterogeneity in the use of additional non-surgical therapies.^14^ In the present study, only 30.4% of the LRCC-M0 and 26.3% of the LRCC-M1 patients who underwent LRCC resection received additional non-surgical therapy, which is on the low end of rates reported in the literature (17–61%).^14^ Only two previous studies investigated the effect from the addition of non-surgical additional therapy on survival outcomes and found no significant effect.^24,25^ It is therefore difficult to come to clear guidelines for the treatment of LRCC, and multimodality treatment remains predominantly based on multidisciplinary decisions.

Although this is the largest study on the incidence and treatment of LRCC, it had several limitations. Due to the timeline of the study, the median clinical follow-up period after resection of the primary colon cancer was only 43 months. Therefore, no 5-year cumulative incidence rates could be presented. However, because most recurrences occur within the first 3 years of colorectal cancer follow-up,^5^ the added value of the 3-year recurrence rates should not be underestimated.^20^

In addition, the site or number of recurrences was not structurally registered in the NCR. Therefore, it was impossible to describe the exact location of the LRCC or to assess possible associations of location on the outcomes. Nonetheless, this study is one of three population-based studies that focused on LRCC and the first in more than a decade. Additionally, the cumulative incidence, risk factors, treatment, and OS of LRCC were analyzed in a single population of primary colon cancer patients, which is unprecedented.

Finally, the stratification between LRCC-M0 and LRCC-M1 and curative-intent treatment, palliative treatment, and best supportive care contributes to the clinical relevance of the analyses performed in this study.

## Conclusions

In conclusion, after diagnosis of primary colon cancer in the first half of 2015, the cumulative incidence of LRCC in the Netherlands was low. Two thirds of LRCC patients presented with synchronous distant metastases. The factors associated with an increased risk of LRCC were age of 70 years or older at disease onset, pT4, pN1-2, and microscopically incomplete resection margins of primary colon cancer. Adjuvant chemotherapy was associated with a lower risk for LRCC. Curative-intent treatment was given to nearly 1 in 4 LRCC patients, resulting in an excellent 3-year OS of 71%, and should always be discussed in multidisciplinary meetings.

## Supplementary Information

Below is the link to the electronic supplementary material.

Supplementary file1 (DOCX 18 KB)
