# Supplementary material for: Locoregionally Recurrent Colon Cancer: How Far Have We Come? A Population-Based, Retrospective Cohort Study
Source: Ann Surg Oncol. 2022 Oct 19;30(3):1726–34. doi: 10.1245/s10434-022-12689-5 (PMC9908679; doi:10.1245/s10434-022-12689-5)
Supplement: Supplementary file 1 — Supplementary file1 (DOCX 18 KB) [file 10434_2022_12689_MOESM1_ESM.docx]

**SUPPLEMENTARY MATERIAL**

**Supplementary table 1 – Univariable and multivariable competing risk regression output for the risk of LRCC according to the cause-specific hazard method.**

|  | 3-year LRCC estimate, % | Univariable  HR (95% CI),  N=3,554 | *P* | Multivariable  HR (95% CI),  N=2,807 | *P* |
| --- | --- | --- | --- | --- | --- |
| Age |  |  |  |  |  |
| <70 years | 2.9 | reference |  | reference |  |
| ≥70 years | 4.7 | 1.7 (1.2; 2.4) | 0.002 | 1.5 (0.99; 2.2) | 0.054 |
| Sex |  |  |  |  |  |
| Male | 3.7 | reference |  |  |  |
| Female | 3.8 | 1.0 (0.72; 1.4) | 0.960 |  |  |
| Location |  |  |  |  |  |
| Right colon | 4.2 | reference |  |  |  |
| Left colon | 3.5 | 0.81 (0.58; 1.1) | 0.228 |  |  |
| Pathological T stage |  |  |  |  |  |
| pT1-3 | 2.2 | reference |  | reference |  |
| pT4 | 13.3 | 7.2 (5.1; 10.1) | <0.001 | 3.8 (2.5; 5.9) | <0.001 |
| Pathological N stage |  |  |  |  |  |
| pN0 | 1.8 | reference |  | reference |  |
| pN1-2 | 7.0 | 4.2 (2.9; 6.0) | <0.001 | 2.0 (1.3; 3.1) | 0.002 |
| Resection margin |  |  |  |  |  |
| R0 | 3.2 | reference |  | reference |  |
| R1-2 | 32.1 | 14.3 (8.6; 23.8) | <0.001 | 5.6 (3.1; 10) | <0.001 |
| No. of assessed lymph nodes |  |  |  |  |  |
| <10 | 3.1 | reference |  |  |  |
| ≥10 | 3.8 | 1.3 (0.59; 2.7) | 0.537 |  |  |
| Morphology |  |  |  |  |  |
| Non-mucinous adenocarcinoma | 3.6 | reference |  |  |  |
| Mucinous adenocarcinoma, signet cell carcinoma and other | 4.8 | 1.3 (0.83; 2.1) | 0.232 |  |  |
| Differentiation grade |  |  |  |  |  |
| Good-moderate | 3.0 | reference |  | reference |  |
| Moderate-no | 8.8 | 3.5 (2.3; 5.3) | <0.001 | 2.0 (1.2; 3.1) | 0.004 |
| Lymphovascular invasion |  |  |  |  |  |
| No | 2.6 | reference |  | reference |  |
| Yes | 7.6 | 3.3 (2.3; 4.7) | <0.001 | 1.9 (1.3; 2.9) | 0.002 |
|  |  |  |  |  |  |

Covariate selection for the multivariable model was based on p<0.10 in univariable analyses. P-values <0.05 were regarded as statistically significant. HR; hazard ratio. CI; confidence interval.
